# Knockdown of *AKT3* (*PKB* **γ**) and *PI3KCA* Suppresses Cell Viability and Proliferation and Induces the Apoptosis of Glioblastoma Multiforme T98G Cells

**DOI:** 10.1155/2014/768181

**Published:** 2014-05-22

**Authors:** Monika Paul-Samojedny, Renata Suchanek, Paulina Borkowska, Adam Pudełko, Aleksander Owczarek, Małgorzata Kowalczyk, Grzegorz Machnik, Anna Fila-Daniłow, Jan Kowalski

**Affiliations:** ^1^Department of Medical Genetics, Medical University of Silesia, 40-055 Katowice, Poland; ^2^Department of Clinical Chemistry and Laboratory Diagnostics, Medical University of Silesia, 40-055 Katowice, Poland; ^3^Division of Statistics, Department of Instrumental Analysis, Medical University of Silesia, 40-055 Katowice, Poland; ^4^Department of Pharmacology, Medical University of Silesia, 40-055 Katowice, Poland

## Abstract

Glioblastoma multiforme (GBM) is the most malignant and invasive human brain tumor that is difficult to treat and has a very poor prognosis. Thus, new therapeutic strategies that target GBM are urgently needed. The PI3K/AKT/PTEN signaling pathway is frequently deregulated in a wide range of cancers. The present study was designed to examine the inhibitory effect of *AKT3* or *PI3KCA* siRNAs on GBM cell growth, viability, and proliferation.T98G cells were transfected with *AKT3* and/or *PI3KCA* siRNAs. AKT3 and PI3KCA protein-positive cells were identified using FC and Western blotting. The influence of specific siRNAs on T98G cell viability, proliferation, cell cycle, and apoptosis was evaluated as well using FC. Alterations in the mRNA expression of *AKT3*, *PI3KCA*, and apoptosis-related genes were analyzed using QRT-PCR. Knockdown of *AKT3* and/or *PI3KCA* genes in T98G cells led to a significant reduction in cell viability, the accumulation of subG1-phase cells and, a reduced fraction of cells in the S and G2/M phases. Additionally, statistically significant differences in the BAX/BCL-2 ratio and an increased percentage of apoptotic cells were found. The siRNA-induced *AKT3* and *PI3KCA* mRNA knockdown may offer a novel therapeutic strategy to control the growth of human GBM cells.

## 1. Introduction

Chemotherapy is the most common therapeutic approach that is used to treat various cancers, but many patients with different cancers (e.g., glioblastoma multiforme) develop chemoresistance. Glioblastoma multiforme (GBM; WHO grade IV) is the most common malignant central nervous system (CNS) tumor and is also the most aggressive form of human astrocytoma [1, 2] with a poor survival rate (approximately 15 months in patients with newly diagnosed cancers regardless of their treatment methods) [3]. The current treatment strategies for GBM that use surgery, chemotherapy, and/or radiotherapy are ineffective and therefore have triggered great research efforts worldwide for new treatment modalities that might be applicable to this cancer.

The PI3 K/AKT pathway, which is an important factor for cell proliferation, growth, survival, invasiveness, and radiation resistance, is critical in the malignant phenotype of GBM [4]. The constitutive or increased activity of the PI3 K/AKT-dependent signaling cascade has been observed in many tumor cells that achieve uncontrolled proliferation. Among the various survival pathways, the PI3K/AKT signaling pathway is often found to be active and plays an important role in the development of GBM.

PI3Ks (phosphoinositide 3-kinases) constitute a family of lipid kinases that are capable of phosphorylating the 3′OH of the inositol ring in phosphoinositides. PI3Ks are divided into three classes according to their structure and function. Class I consists of two subclasses—class IA and class IB, respectively. Class IA includes heterodimers that are composed of a p110 catalytic subunit and a p85 regulatory subunit. A p110 subunit has three isoforms (p110*α*, p110*β*, and p110*γ*) that are encoded by the three different genes [5–8]. These isoforms are involved in the regulation of processes such as proliferation, cell survival, degranulation, vesicular trafficking, and cell migration. After activation, the p110 subunit phosphorylates the PIP_2_ (phosphatidylinositol-4,5-biphosphate) into PIP_3_    (phosphatidylinositol-3,4,5-triphosphate) [9]. Similar to Class IA, Class IB includes heterodimers that are composed of a catalytic subunit p110*γ* and a regulatory subunit p101. Two new regulatory subunits (p84 and p87PIKAP) have also been described by some authors [10]. Class II consists of single catalytic subunits (isoforms PI3KC2*α*, PI3KC2*β*, and PI3KC2*γ*). Finally, class III involves a single catalytic subunit Vps34. The p110*α* catalytic subunit of PI3K is encoded by the* PI3KCA* gene (locus 3q26.3) [7, 11]. The activity of a p110*α* subunit of PI3K is regulated by a p85 subunit [12]. It has been suggested that in cells in which the p110*α* isoform of PI3K is predominant or in which both p110*α* and p110*β* isoforms are equally important, the knockdown of* PIK3CA* (p110*α*)   interferes with PI3K/AKT signaling [13]. The* PI3KCA* gene has been found to be amplified and overexpressed in several types of cancers. It has been suggested that the point mutations that activate the* PI3KCA* gene may represent a novel mechanism for the induction oncogenic PI3K signaling pathway [14, 15]. Hafsi et al. [15] stressed the fact that oncogenic* PI3KCA* mutations play a critical role in human malignancies and provide evidence that kinases with cancer-specific mutations such as PI3K may be ideal targets for small-molecule specific inhibitors that would create the opportunity to develop new anticancer drugs [15].* PI3KCA* gene mutations have been found in several cancers (e.g., liver, breast, colorectal, brain, and gastric) and the majority of these have been shown constitutively to activate the protein's catalytic subunit [16, 17]. The point mutations that activate the* PIK3CA* have been observed in some gliomas [18]. In a few cases of GBMs, cell proliferation is specifically blocked by the downregulation of p110*α* alone [19]. PI3K recruits AKT into the cell membrane through the PIP_3_ binding domain and allows PDK1 (3-phosphoinositide-dependent kinase) to activate AKT through the phosphorylation of AKT at T308 position and the activation of its serine/threonine kinase activity [20].

It was also found that GBM often upregulates the PI3K signaling pathway through the loss of PTEN or through the activation of receptor tyrosine kinases (RTKs) [21]. The AKT kinase plays an important role in the PI3K signaling pathway as it is one of the major downstream effectors. The activity of AKT is induced following PI3K activation in various growth factor receptor-mediated signaling cascades [22]. AKT (PKB, RAC-PK) is a serine/threonine protein kinase that is involved in the regulation of many cellular processes such as growth, intermediate metabolism, survival, proliferation, invasiveness, and the regulation of tumor angiogenesis. AKT is the key regulator of different cellular functions acting via the phosphorylation of a variety of substrates. For example, AKT inhibits apoptosis through the inactivation of BAD, which is a proapoptotic member of the BCL-2 protein family [23, 24], as well as by the phosphorylation of caspase-9 [25] or by inhibiting the Forkhead transcription factors [26, 27]. AKT is also involved in the regulation of the cell cycle and cell proliferation [28]. There are three isoforms of AKT (AKT1, AKT2, and AKT3), which are encoded by three different genes. The AKT2 and AKT3 (but not AKT1) isoforms are pathologically amplified in human cancers [29, 30]. AKT3 tissue distribution is more restricted than AKT1 and AKT2; it is primarily expressed in the brain and testis [31]. An increase in the* AKT3* mRNA level has been found in breast and prostate cancers [29]. It is also known that AKT2 and AKT3 are overexpressed in glioma cells and play a pivotal role in malignant gliomas [4]. The activation of AKT has been found in approximately 80% of human GBMs [32–37]. The increased AKT activity in tumors may be a result of* PI3K* gene amplification. As was mentioned earlier, AKT is activated via the receptor tyrosine kinases in a PI3K-dependent manner. Several studies have suggested that AKT activation correlates with a resistance to radio- and chemotherapy [6].

The current knowledge concerning the molecular mechanisms of GBM development indicates that PI3K kinase is a very promising target for therapy. Some authors have suggested that PI3K activation is associated with the chemoresistance of GBM cells, that is, with the lack of sensitivity to various chemotherapeutic agents [15]. Therefore, it is very important to discover the exact mechanisms that determine the PI3K/AKT signaling activity and to understand how the inhibition of this pathway influences the main cellular processes. We hope that our research will help find a way to inhibit PI3K/AKT signaling so that it can be used in routine clinical practice.

In the present study, AKT3 (PKB*γ*) and PI3KCA (p110*α*) were targeted with siRNAs in order to examine the inhibition of their signaling cascade on the growth, viability, proliferation of glioblastoma multiforme, and the induction of apoptosis. We decided to knockdown only the gene that encodes the p110*α* subunit because it plays a crucial role in tumorigenesis. All analyses were conducted using the T98G cell line. This particular cell line was chosen because it is less sensitive to BCNU (bis-chloroethylnitrosourea; carmustine) and etoposide than the U87-MG cell line [38]. Moreover, the T98G cell line shows an increased resistance to temozolomide, compared to other cell lines such as U373-MG, U251-MG, GB-1, U87-MG, or A-172 [39]. Thus, it seems reasonable to use this particular cell line when searching for new potential therapeutic methods of glioma therapy. We also investigated whether the knockdown of* AKT3* and* PI3KCA* genes plays a role in the induction of apoptosis and the reduction of cell viability and proliferation.

Our findings demonstrate for the first time that the siRNAs that target* AKT3* and* PI3KCA* reduce cell viability and induce apoptosis in T98G cells. Thus, the knockdown of* AKT3* and* PI3KCA* genes may offer a potential therapeutic solution for controlling the growth of human glioblastoma multiforme cells.

## 2. Material and Methods

### 2.1. Cell Cultures

The T98G cell line, which was derived from a 61-year-old male [27] and purchased from American Type Culture Collection (ATCC, Manassas, VA, USA), was cultured in modified Eagle's minimum Essential Medium (ATCC) supplemented with heat-inactivated 10% fetal bovine serum (ATCC) and 10 *μ*g/mL gentamicin (Invitrogen). The cell line was maintained at 37°C in a humidified atmosphere of 5% CO_2_ in air.

### 2.2. siRNA Transfection

**Figure 1:**
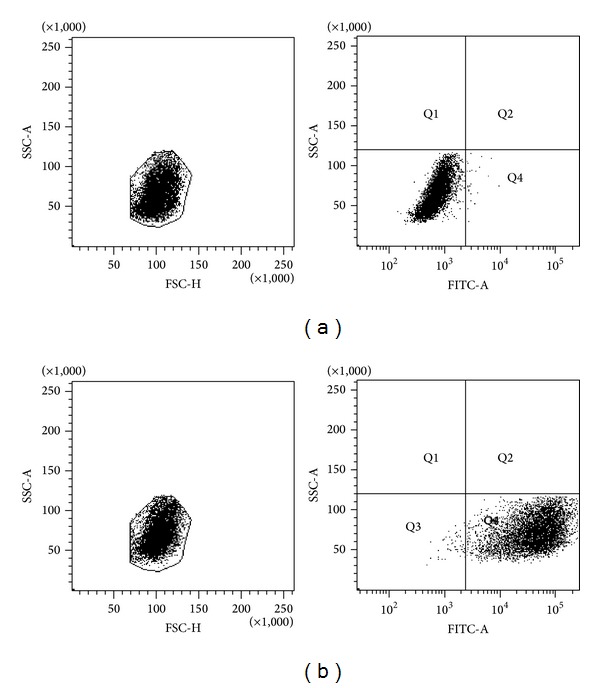
Transfection efficiency of T98G cells as assessed by flow cytometry ((a) untransfected cells; (b) transfected cells) 24 h after transfection with 1 nM siRNA labeled at the 3′ end of the sense strand with AlexaFluor488 (green fluorescence).

T98G cells were seeded at 1.6 × 10^4^ cells per well in 6-well plates and incubated for 24 h. Next, the T98G cell line was transfected with specific siRNAs that target* AKT3* or* PI3KCA* mRNA. Transfection was performed using the FlexiTube siRNA Premix (Qiagen, Italy) according to the manufacturer's protocol. The following target sequences were used: 5′ AACTGTTGGCTTTGGATTAAA 3′ (for* AKT3*) and 5′ CTGAGTCAGTATAAGTATATA 3′ (for* PI3KCA*). The knockdown of* AKT3* and* PI3KCA* was provided with specific siRNAs without affecting* AKT1* and* AKT2* mRNAs. Optimum transfection conditions were established by using various amounts of the FlexiTube siRNA Premix (25 nM, 10 nM, 5 nM, 1 nM, and 0.5 nM) and the number of cells (1–4 × 10^4^ cells per well in 6-well plates). The optimal conditions were 1 nM siRNA for* AKT3* and* PIK3CA* and a 48 h incubation time. After transfection, prior to performing assays, the cells were washed with PBS, trypsinized, and centrifuged (125 g/5 min) at 4°C (listed below). Transfection efficiency was checked using flow cytometry (Figures 1(a) and 1(b)) and fluorescence microscopy using siRNA labeled with AlexaFluor488. The results are representative of at least three independent experiments.

The average transfection efficiency was 99.2% (Figure 1(b)).

AllStars Negative Control siRNA was also tested (Qiagen, Italy). This siRNA has no homology to any known mammalian gene and a variety of cell-based assays have shown its minimal nonspecific effect on gene expression and phenotype.

### 2.3. RNA Extraction

Total RNA was isolated from cultured cells using the TRIzol reagent (Life Technologies, Inc., Grand Island, NY, USA) according to the manufacturer's protocol. The integrity of total RNA was checked using electrophoresis in 1% agarose gel stained with ethidium bromide. All RNA extracts were treated with* DNAse I* to avoid genomic DNA contamination and were assessed qualitatively and quantitatively.

### 2.4. The Evaluation of Transcriptional Activity of AKT3, PI3KCA, BCL-2, and BAX Using QRT-PCR

QRT-PCR assays were performed using an ABI Prism7700 (Applied Biosystems, Foster City, USA). Real-time fluorescent RT-PCR was performed using a TaqMan Gene Expression Assay (manufacturer does not provide the primers' sequence) and a TaqMan One-Step RT-PCR Master Mix Reagents Kit (Applied Biosystems) according to the manufacturer's protocol under the following conditions: 48°C for 30 min and 95°C for 10 min, followed by 40 cycles of 15 sec at 95°C and 1 min at 60°C. RNA for human glyceraldehyde-3-phosphate dehydrogenase (GAPDH) was used as an endogenous control. The copy numbers for each sample were calculated using the *C*_*T*_-based calibrated standard curve method. Data were normalized to *β*-actin. Each data point is the average of duplicates.

### 2.5. The Evaluation of AKT3 and PI3KCA Protein Expressions Using Flow Cytometry

AKT3 protein-positive cells were identified using indirect labeling with a specific anti-AKT3 antibody and flow cytometry. After the* AKT3* knockdown, cells were harvested, washed twice in PBS, and fixed in PBS with 4% paraformaldehyde and 10% goat serum. The cells were then washed twice in PBS with 1% BSA, permeabilized with 0.1% saponin/1% BSA/PBS with 10% goat serum for 45 min, and incubated with 10 *μ*g/mL of an anti-AKT3 monoclonal antibody (isotype IgG_2A_, R&D Systems, Inc.) in 1% BSA/PBS overnight at 4°C. Subsequently, cells were washed followed by the addition of antimouse secondary antibodies conjugated with FITC for 45 min and analyzed quickly using flow cytometry.

PI3KCA protein-positive cells were identified using direct labeling with a specific anti-PI3KCA antibody and flow cytometry. After* PI3KCA* knockdown, cells were harvested, washed twice in PBS, and fixed in PBS with 4% paraformaldehyde. Then, the cells were washed twice in PBS with 1% BSA, permeabilized with 0.1% saponin/1% BSA/PBS for 45 min, and incubated overnight at 4°C with 10 *μ*g/mL of an anti-PI3KCA polyclonal antibody conjugated with PE (Bioss) in 1% BSA/PBS. An isotype-matched monoclonal antibody (isotype control) was used to determine nonspecific binding. Subsequently, the cells were washed and analyzed using a FACSAria (BD Biosciences) equipped with Diva Software.

### 2.6. Western Blot Analysis of AKT3 and PI3KCA Proteins

Six-well plates with both transfected and untransfected T98G cells were placed on ice, washed with ice-cold PBS, harvested by scraping, and stored in a cold RIPA buffer 1x PBS, 1% Triton X-100, 0.5% sodium deoxycholate, 0.1% SDS, 1 mM Na_3_VO_4_, 1 mM NaF, 1*μ*M okadaic acid, and 10 mM *β*-glycerolphosphate supplemented with a protease inhibitor cocktail (Complete minitablets, Roche, Indianapolis, IN, USA). The collected material was then incubated on ice for 30 min with frequent gentle vortexing. The lysates were cleared by centrifugation at 13000 g for 15 min. The total protein concentration in the samples was measured using Bradford method and an xMark Microplate Spectrophotometer (BioRad, CA, USA). Bovine serum albumin of known concentrations within the range of 0–1500 ng/*μ*L (Thermo Fermentas, Lithuania) were used to prepare the calibration curve. Equal amounts of protein (30 *μ*g) from cell lysates were resolved on 10% SDS-polyacrylamide gels and then transferred onto PVDF membranes at room temperature overnight. Blots were blocked for 1 h with 1% BSA in Tris-buffered saline containing 0.1% Tween-20 (TBS-T) (Sigma-Aldrich) and probed for 1.5 h at room temperature with the appropriate primary antibody in TBS-T containing 1% BSA (affinity-purified mouse polyclonal anti-AKT3; 1 : 1000 dilution; R&D Systems Inc., or affinity-purified rabbit polyclonal anti-PI3KCA; 1 : 1000 dilution; Bioss, MA, USA). Antibodies against *β*-actin (Abcam) were used as the loading control for the samples and for further estimating the relative gene expression levels. After washing with TBS-T, the blots were incubated for 1 h at room temperature with the appropriate horseradish peroxidase-conjugated antimouse or antirabbit IgG secondary antibody (GE Healthcare) in TBS-T containing 1% BSA and then washed 1x in TBS-T and 1x in TBS, respectively. Protein bands were detected using enhanced chemiluminescence (ChemiDoc-It Imaging System). Scanning densitometry (ImageI) was done in order to quantify band intensities by volume/area integration.

### 2.7. Viability and Proliferation Assays

The effects of* AKT3, PI3KCA,* and* AKT3* +* PI3KCA* silencing on T98G cell proliferation and viability were estimated using the cell proliferation reagent WST-1 (Roche) and by the direct counting of cells that had been stained with trypan blue. The WST-1 assay was based on the cleavage of the tetrazolium salt WST-1 to formazan by cellular mitochondrial dehydrogenases. An increase in the overall activity of mitochondrial dehydrogenases in a sample is linked to an increase in the number of viable cells. The formazan dye formation was quantified using a plate reader at 450 nm. T98G cells were plated onto a 96-well microplate at a density of 1 × 10^4^ cells per well. The cells were then transfected with 1 nM* AKT3*,* PI3KCA*, or* AKT* +* PI3KCA* siRNAs (a few wells were left untransfected). The negative control wells received serum-free media. Cells were allowed to incubate for 48 h before the WST-1 reagent was added and then incubated for 1 h in order to measure cell proliferation (in tetraplicate). Data are presented as a percentage of the proliferation of the negative control. The percentage viability was calculated considering the controls as 100%. A *P* value < 0.05 was considered to be significant. Each experiment was repeated six times.

Cell viability was also measured using a trypan blue exclusion assay. Harvested cells were mixed with an equal amount of trypan blue (10 *μ*L) and after staining, the cells were counted using a Bürker chamber. The T98G cell viability was calculated as the percentage of live cells in the total cell population.

### 2.8. Cell Cycle Analysis

T98G cells were seeded in 6-well plates (at a density of 1.6 × 10^4^ cells per well) and cultured overnight (24 h) and, after a medium exchange, they were further cultured for 48 h in the presence of* AKT3*,* PI3KCA*, and* AKT3* +* PI3KCA* siRNAs (final concentration 1 nM). Fewer than 90% of the confluent cells were trypsinized and washed twice with ice-cold phosphate-buffered saline (PBS), fixed in 70% ice-cold ethanol while undergoing low-speed vortexing (incubation for 1 h on ice). The samples were then treated with RNAse A (10 mg/mL; for 1 h at 37°C); nuclei were stained with propidium iodide (PI, 50 *μ*g/mL) and analyzed using flow cytometry (FACS AriaII, Becton Dickinson). DNA histograms of PI-stained cells and histograms showing the distribution of cells in the different phases of the cell cycle were assessed. A total of 1 × 10^4^ nuclei from each sample were analyzed on a FACS AriaII flow cytometer and DNA histograms were examined using BD FACSDiva software (Becton Dickinson). A gating strategy based on forward scatter versus side scatter was used to exclude doublets and debris.

### 2.9. Apoptosis Assay

T98G cells were seeded in 6-well plates (at a density of 1.6 × 10^4^ cells per well) and cultured overnight (24 h) and, after a medium exchange, they were further cultured for 48 h in the presence of* AKT3*,* PI3KCA*, or* AKT3* +* PI3KCA* siRNAs (final concentration 1 nM). After trypsinization and washing twice with Hank's Balanced Salt Solution (HBSS), the cells were suspended in 1 mL of HBSS. In the next step, the cells were analyzed using flow cytometry with a Vybrant DyeCycle Violet/SYTOX AADvanced Apoptosis Kit. The staining pattern that resulted from the simultaneous use of both dyes in combination make it possible to distinguish normal, apoptotic, and necrotic cell populations as described in the manufacturer's protocol.

### 2.10. Statistical Analysis

The data that was generated by the WST-1 assay are presented as mean ± SD.* t*-Student test and the* U* Mann-Whitney test or Kruskal-Wallis ANOVA was used for comparing two or more groups. The power of all of the tests was not less than *β* = 0.8. Data were analyzed using Statistica software (StatSoft, Inc. 2008) version 9.0 (http://www.statsoft.com/). All of the tests were two-sided and *P* < 0.05 was considered to be statistically significant. The proliferation and apoptotic indexes were determined according to Darzynkiewicz et al. [40] and Henry et al. [41].

## 3. Results

### 3.1. AKT3 and PI3KCA siRNAs Decrease the Copy Numbers of Particular mRNA and AKT3 PI3KCA Protein Levels in T98G Cells

**Figure 2:**
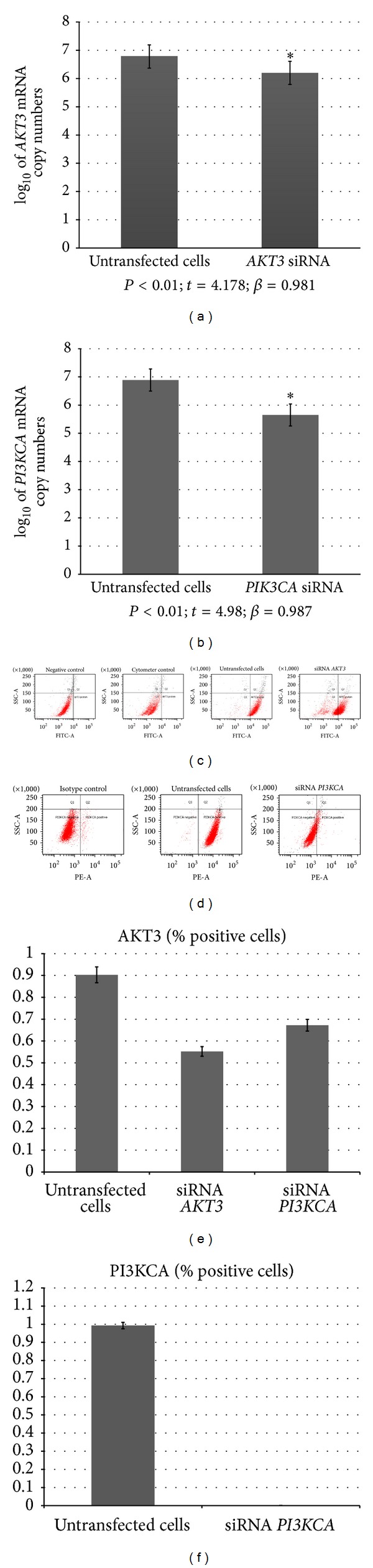
Effect of siRNA on T98G cells. ((a), (b)) Comparison of mRNA copy numbers for* AKT3* (a) and* PI3KCA* (b) between transfected and untransfected T98G cells (*AKT3*: *P* < 0.01, *t* = 4.178, *β* = 0.981;* PI3KCA*: *P* < 0.01, *t* = 4.98, *β* = 0.987); ((c), (d)) dot-plots for comparison of the percentage of AKT3 protein-positive cells after* AKT3* and* PI3KCA* knockdown and without using* AKT3* siRNA; analysis was performed using flow cytometry; ((e), (f)) comparison of the percentage of AKT3 or PI3KCA protein-positive cells between the groups that were analyzed (*P* < 0.05).

The* AKT3* and* PIKCA* mRNA copy numbers were quantified in transfected and untransfected T98G cells, respectively. We found significantly lower mRNA levels of both* AKT3* and* PI3KCA* in the transfected cells compared to the untransfected (control) cells (Figures 2(a) and 2(b)).

AKT3 and PI3KCA protein levels were examined in transfected and untransfected T98G cells using flow cytometry. We found a significantly lower percentage of AKT3-positive cells following transfection with* AKT3* siRNA, or* PI3KCA* siRNA, as well as PI3KCA-positive cells after transfection with* PI3KCA* siRNA compared to the untransfected cells (Figures 2(c), 2(d), 2(e), and 2(f)).

**Figure 3:**
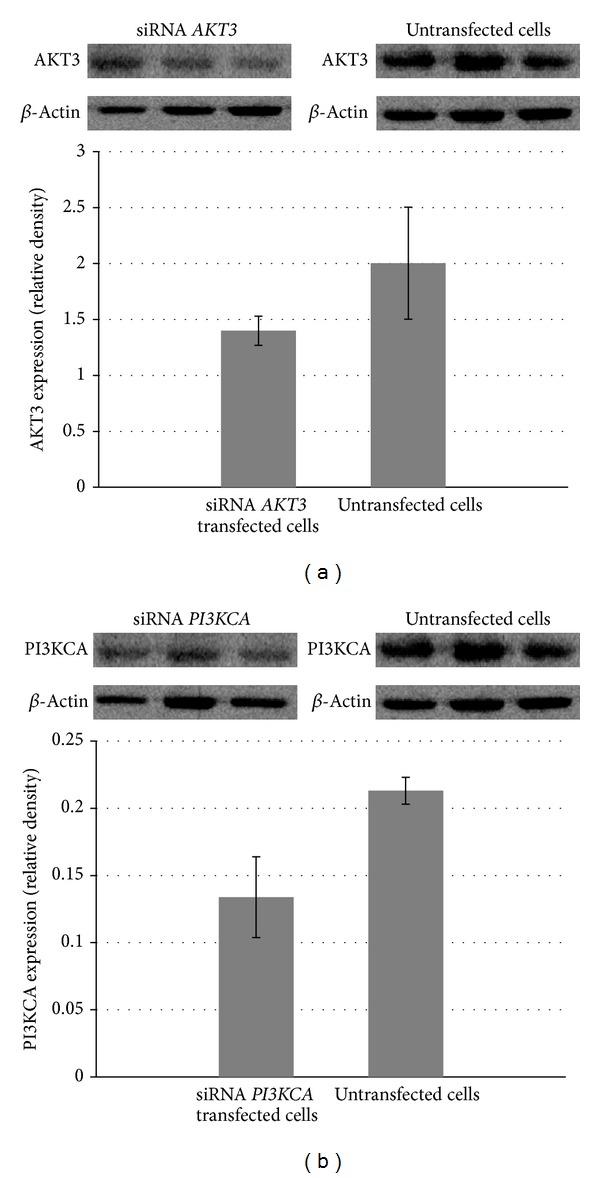
Reduction in the AKT3 and PI3KCA protein expression in T98G cells after* AKT3* and* PI3KCA* knockdown compared to untransfected cells. All of the figures are representative of three independent experiments. Data are expressed as means ± SEM (*P* < 0.05).

Changes in the expression of AKT3 and PI3KCA proteins were also analyzed using Western blot technique. Densitometric analysis of AKT3 and PI3KCA bands showed that protein levels were markedly reduced in the T98G cells after transfection with siRNAs that were specific for* AKT3* or* PI3KCA* genes, respectively (Figure 3; *P* < 0.05).

### 3.2. AKT3 and PI3KCA siRNAs Decrease Cell Viability in T98G Cells

In the next step, we examined the effect of* AKT3*,* PI3KCA*, and* AKT3* +* PI3KCA* siRNAs on the proliferation and viability of T98G cells using the WST-1 reagent and trypan blue staining. The results are expressed as a percentage of the viability of the control cells (arbitrarily assigned 100% viability). Transfection of T98G cells with* PI3KCA* and* AKT3* +* PI3KCA* siRNAs led to a significant reduction in cell viability compared to the untransfected controls. The simultaneous transfection of T98G cells with siRNAs that target* AKT3* and* PI3KCA* did not provide an additive cytotoxic effect as was expected.

**Figure 4:**
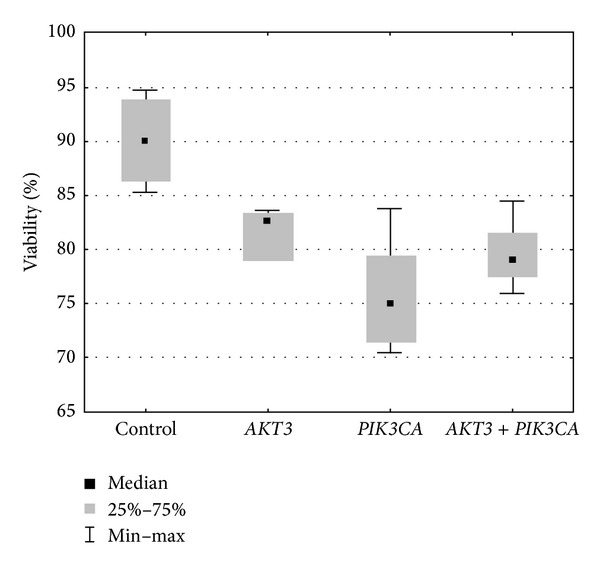
Cytotoxic effect of* AKT3* and* PI3KCA* siRNAs on T98G cell viability as determined by trypan blue exclusion and the WST-1 assay (*P* < 0.05).

Data (collected from 14 independent experiments) are represented as the median ± S.D and shown as a so-called “box-plot” with the median, lower, and upper quartiles as boxes and the min-max values as lines (Figure 4; *P* < 0.05, compared to the control cells).

### 3.3. AKT3, PI3KCA, and AKT3 + PI3KCA siRNAs Decrease the Number of T98G Cells in the S and G2/M Phases and Decrease the DNA and Proliferation Indexes

**Figure 5:**
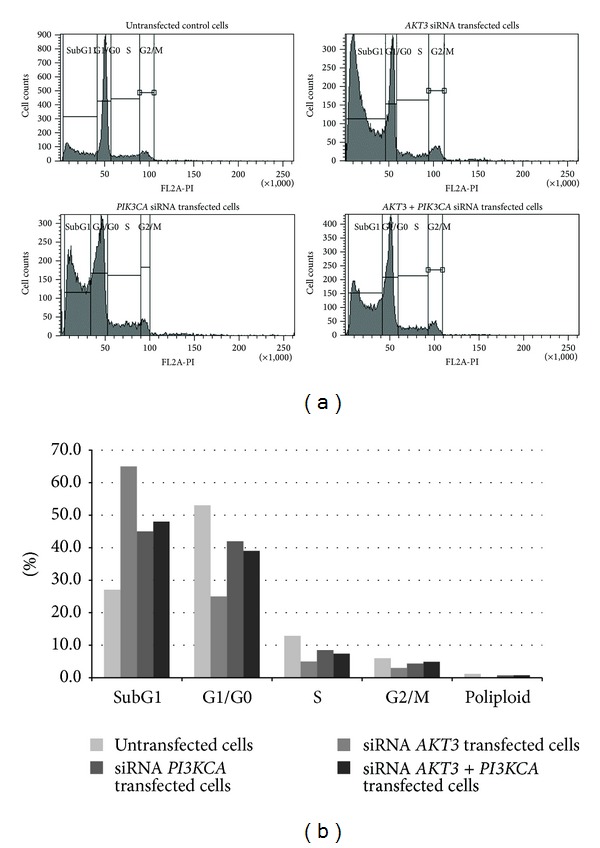
(a) DNA content frequency histograms representing cells from untransfected cultures and from cultures that had been transfected with* AKT3*,* PI3KCA*, and* AKT3* +* PI3KCA* siRNAs. (b) The cell cycle distribution of the T98G cell culture that had been transfected with* AKT3*,* PI3KCA*, and* AKT3* +* PI3KCA* siRNAs, respectively, compared to the untransfected control cells. Cells were fixed in 70% ethanol and stained with PI. Fluorescence of the PI-stained cells was measured using a FACS AriaII cytometer (Becton Dickinson).

**Figure 6:**
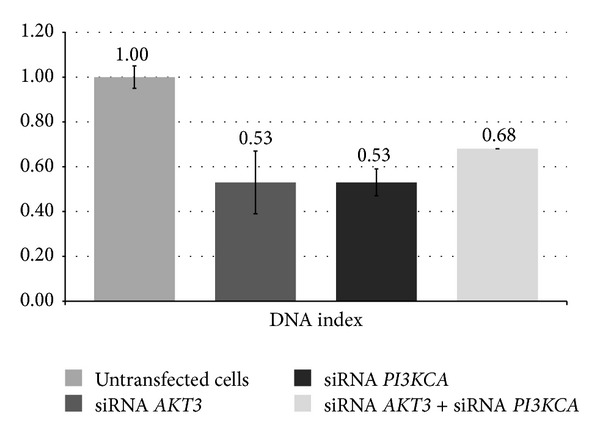
Comparison of the DNA index (the ratio of transfected cell DNA fluorescence/untransfected G1-G0 cell DNA fluorescence) between cells that had been transfected with* AKT3*,* PI3KCA*, and* AKT3* +* PI3KCA* siRNAs and untransfected cells (*P* < 0.05). A DNA index of one corresponds to diploidy (2C).

In order to examine the possible mechanisms of the antiproliferative activity of* AKT3* and* PI3KCA* siRNAs, cell cycle distribution using flow cytometry was performed. Proliferation index (PI), that is, percentage of proliferating cells in S + G2/M cell cycle phases, was determined. PI was quantified in untransfected T98G cells and after the knockdown with adequate 1 nM siRNA. It was found that* AKT*,* PI3KCA*, and AKT3 +* PI3KCA* siRNAs decrease the percentage of cells in the S + G2/M phases (lower PI) and simultaneously increase the percentage of cells in subG1 phase, as compared to untransfected (control) cells (Figures 5 and 6, resp.).

**Figure 7:**
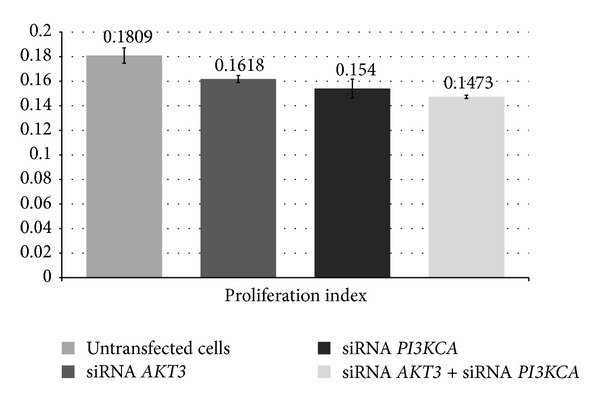
Comparison of the proliferation index between cells that had been transfected with* AKT3*,* PI3KCA,* or* AKT3* +* PI3KCA* siRNAs and untransfected cells (*P* < 0.05).

We also found that* AKT3* and* PI3KCA* siRNAs decrease the DNA index (the ratio of transfected cell DNA fluorescence/untransfected G1-G0 cell DNA fluorescence) (Figures 6 and 7; *P* < 0.05 compared to the control cells).

### 3.4. AKT3 and PI3KCA siRNAs Decrease the Expression of the BCL-2 mRNA and Increase the Expression of the BAX mRNA in T98G Cells

The BCL-2 family includes important regulators of apoptotic cell death. It is known that the ratio of BAX/BCL-2 is critical for the induction of apoptosis [42]. The BAX protein is regulated by AKT through phosphorylation that occurs near the C-terminus at serine 184 (direct control) or by GSK-3 kinase (indirect regulation) [33]. Therefore, we also examined the changes in the levels of the* BCL-2* and* BAX* mRNAs expression in transfected and untransfected T98G cells.

**Figure 8:**
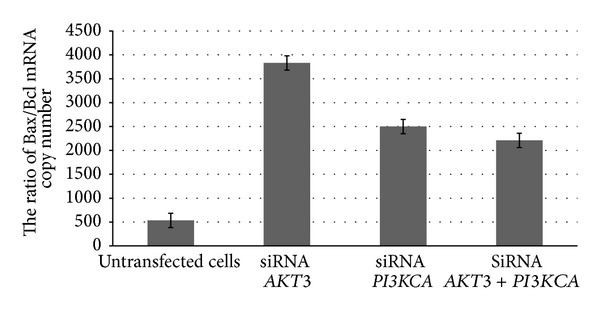
BAX/BCL-2 ratio in T98G cells after* AKT3*,* PI3KCA*, and* AKT3* +* PI3KCA* gene silencing using specific siRNAs (*P* < 0.05).

A statistically significant increase in the* BAX/BCL-2* mRNAs level ratio was observed after the siRNA-mediated silencing of* AKT3*,* PI3KCA*, and* AKT3* +* PI3KCA* genes in transfected cells compared to untransfected control cells (Figure 8).

### 3.5. AKT3, PI3KCA, and AKT3 + PI3KCA siRNAs Can Induce Apoptosis

**Figure 9:**
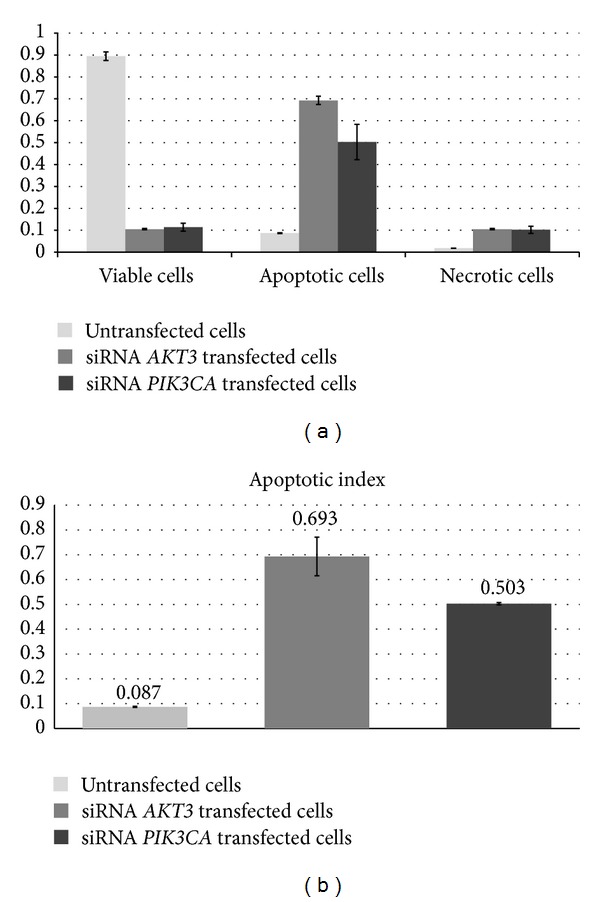
The influence of* AKT3* and* PI3KCA* gene knockdown on the induction of apoptosis. The datasheets show the apoptosis and necrosis rates in the groups that were analyzed (a) and the value of the apoptotic index (b). The data are expressed as the means of three separate experiments (*P* < 0.05).

Necrotic and apoptotic cells were detected using flow cytometry with double staining with Vybrant DyeCycle Violet and SYTOX AADvanced following transfection with* AKT3* and* PI3KCA* siRNAs. The knockdown of* AKT3* and* PI3KCA* genes led to apoptosis in 69.3% cells (*AKT3* siRNA) and 50.3% cells (*PI3KCA* siRNA), respectively, compared to 8.7% in the untransfected control cells (Figure 9(b); *P* < 0.05). In contrast, the necrosis rates of transfected T98G cells were 19.7% and 10.2% after* AKT3* and* PI3KCA* silencing, respectively, whereas the necrosis rate of the untransfected control cells was only 1.8% (Figure 9(a); *P* < 0.05). We observed a 6- to 8-fold higher apoptotic index value after cell transfection with siRNAs (Figure 9(b); *P* < 0.05).

## 4. Discussion

More effective therapies for the treatment of CNS neoplasms are urgently needed. The present study was focused on the cell cycle, apoptotic behavior, and viability of the human glioblastoma multiforme T98G cell line. The PI3K/AKT signaling pathway plays a crucial role in carcinogenesis and the development, progression, and invasiveness of GBM.

Before we began to study the changes in the cellular processes in question, we verified whether transfection with a given siRNA did indeed silence the respective gene expression. As we expected, the expression levels of* AKT3* and* PI3KCA* mRNAs were significantly downregulated after transfection with specific siRNAs. This was also confirmed by Western blot and flow cytometry analyses. Both analyses showed a reduction in the AKT3 and PI3KCA protein levels in the siRNA-transfected cells compared to the control (untransfected) cells. This step confirmed that transfection of T98G cells with specific siRNAs downregulates the expression of* AKT3* and* PI3KCA* genes.

The PI3K/AKT signaling pathway is responsible for the regulation of cell cycle progression and proliferation. AKT promotes the G1/S phase transition by blocking the FOXO-mediated transcription of cell cycle inhibitors. We found that siRNAs that target* AKT3* and* PI3KCA* decrease the percentage of T98G cells in the S phase and mitosis and increase the percentage of T98G cells that undergo apoptosis (subG1 fraction). We showed that the knockdown of* AKT3* and* PI3KCA* genes triggers a reduction in the proliferative index and inhibits the proliferation of T98G cells. The complex character of proliferative response may explain the relatively small differences between transfected and untransfected cells that were observed in our experiment. Several different pathways are involved in the cell proliferation process (e.g., receptor-initiated signal transduction, kinase activation, target phosphorylation, and DNA synthesis). However, our results as well as previous observations by Weber et al. [1] show that* PI3KCA* knockdown results in decreased GBM cell proliferation. This seems to confirm that the PI3K/AKT pathway is very important in proliferation process.

The activation of AKT3 by IGF-1 suggests that AKT3 may be involved in regulating cell survival [43]. We found that* AKT3*,* PI3KCA*, and* AKT3* +* PI3KCA* silencing by siRNAs significantly reduces the viability of T98G cells. An increase in the percentage of cells in the subG1 phase (apoptotic cells) was accompanied by a reduction in the number of cells in the S and G2/M phases. We suggest that* AKT3* and* PI3KCA* siRNAs induce an intrinsic apoptotic pathway in T98G cells.

AKT plays an important role in regulating apoptosis by phosphorylating and inhibiting the maturation of procaspase 9, a protease that is crucial for the execution phase of apoptosis, or by the phosphorylation and inactivation of BAD as was mentioned before [25]. BAD inhibits the function of the prosurvival molecule BCL-X_L_. AKT also prevents apoptosis through the activation of antiapoptotic proteins such as BCL-2, I*κ*B kinase (IKK) and HDM2. The inhibition of apoptosis depends on the intracellular balance between BCL-2 and BAX activity and other proteins that belong to the BCL-2 family like BAG1, BAD, BCL-X_L_, and BCL-X_S_ [44, 45]. An increase in the BAX/BCL-2 ratio stimulates the release of cytochrome c from the mitochondria into cytosol. Since defects in apoptosis may be essential for building up a resistance to the majority of current treatments, it would be highly desirable to develop a strategy that would reduce the resistance of glioblastoma cells to apoptosis. We found that* AKT3*,* PI3KCA*, and* AKT3* +* PI3KCA* siRNAs increased* BAX* mRNA expression and decreased* BCL-2* mRNA expression, which is compatible with an increase in the* BAX/BCL-2* ratio. Our results suggest that* AKT3* and* PI3KCA* siRNAs have the ability to alter the mRNA expression of the BCL-2 family of apoptosis-related genes. We also found that the knockdown of* AKT3* and* PI3KCA* genes was associated with a significantly higher apoptotic index. Our results are consistent with the findings of previous studies. Opel et al. [46] reported that the inhibition of PI3K is an efficient strategy to sensitize glioblastoma cells to the induction of apoptosis. Koseoglu et al. [47] analyzed the influence of* AKT1*,* AKT2*, and* AKT3* knockdown on the induction of apoptosis in 20 human tumor cell lines and found that the knockdown of* AKT* resulted in apoptosis in six out of 11 tumor cell lines with activated AKT. Stahl et al. [48] recorded that a targeted reduction in the AKT3 expression with siRNA stimulates apoptosis and inhibits the development of melanoma tumors. Finally, Mure et al. [4] observed an induction of apoptosis that was mediated by a mitochondrial pathway through the dephosphorylation of BAD and the activation of caspase 3 and 9 in glioma cell lines that had been exposed to AKT3 knockdown.

To summarize, our study is the first to demonstrate the results of* AKT3* and* PI3KCA* silencing in T98G cells. Unexpectedly, we found that the simultaneous knockdown of* AKT3* and* PI3KCA* has a weaker effect than the silencing of these genes separately. These results may suggest that the simultaneous knockdown of* AKT3* and* PI3KCA* genes leads to a cascade of another signaling pathway that mediates cell cycle and apoptosis regulation. There is another Ras/Raf/MAPK pathway that is known to be extremely important in GMB tumorigenesis in addition to the PI3K/AKT pathway. Sunayama et al. [49] showed that the inactivation of the Ras/Raf/MAPK pathway triggered a reciprocal activation of the PI3K/AKT pathway and vice versa, which suggests that there may be a mutually inhibitory crosstalk between them. It could be that the simultaneous silencing of* PI3KCA* and* AKT3* leads to a greater activation of Ras/Raf/MAPK pathway, but this phenomenon needs to be clarified.

T98G cells are less sensitive to BCNU and etoposide than U87-MG cells [46]. In addition, the T98G cell line is the most resistant to temozolomide compared to cell lines such as U373-MG, U251-MG, GB-1, U87-MG, and A-172 [12], which is a major chemotherapeutic agent in the current treatment of GBM. Thus, it seems reasonable to conduct research for potential new glioma therapy methods using that cell line.

## 5. Conclusions

In the T98G glioblastoma multiforme cell line, the expression of* AKT3* or* PI3KCA* genes is downregulated by siRNAs that target their mRNAs and this leads to the upregulation of the* BAX/BCL-2* mRNA expression ratio. We also showed that* AKT3* and* PIK3CA* siRNAs significantly reduced the respective protein levels. Moreover,* AKT3* and* PI3KCA-*specific siRNAs reduced the viability and proliferation of GBM cells and promoted their apoptosis via mitochondrial pathway. Therefore, blocking* AKT3* and* PI3KCA* with suitable siRNAs offers a potential therapeutic strategy for controlling the growth of human glioblastoma multiforme cells. Further studies are warranted in order to determine whether targeting* AKT3* and* PI3KCA* genes with siRNAs has a true potential for glioblastoma multiforme treatment* in vivo*.
